# Performance Characterization of Broad Band Sustainable Sound Absorbers Made of Almond Skins

**DOI:** 10.3390/ma13235474

**Published:** 2020-12-01

**Authors:** Stefania Liuzzi, Chiara Rubino, Pietro Stefanizzi, Francesco Martellotta

**Affiliations:** Department of Civil Engineering Sciences and Architecture, Polytechnic University of Bari, via Orabona 4, I-70125 Bari, Italy; chiara.rubino@poliba.it (C.R.); pietro.stefanizzi@poliba.it (P.S.); francesco.martellotta@poliba.it (F.M.)

**Keywords:** agro-waste, sound absorption, hygrothermal performances, sustainable materials, circular economy

## Abstract

In order to limit the environmental impact caused by the use of non-renewable resources, a growing research interest is currently being shown in the reuse of agricultural by-products as new raw materials for green building panels. Moreover, the European directives impose the goal of sustainability supporting the investigation of passive solutions for the reduction of energy consumption. Thus, the promotion of innovative building materials for the enhancement of acoustic and thermal insulation of the buildings is an important issue. The aim of the present research was to evaluate the physical, acoustical, and thermal performances of building panels produced by almond skin residues, derived from the industrial processing of almonds. In this paper different mix designs were investigated using polyvinyl acetate glue and gum Arabic solution as binders. Air-flow resistivity σ and normal incidence sound absorption coefficient α were measured by means of a standing wave tube. Thermal conductivity λ, thermal diffusivity α, volumetric heat capacity *ρc* were measured using a transient plane source device. Finally, water vapor permeability *δ_p_* was experimentally determined using the dry cup method. Furthermore, a physical characterization of the specimens in terms of bulk density *ρ_b_* and porosity *η* allowed to study the correlation existing between the binder and the aggregates and the consequent acoustical and hygrothermal behavior occurring on the different mix designs. The achieved results suggested the investigated materials comparable to the main products currently existing on the market.

## 1. Introduction

A stunning growth in the quantity and variety of solid wastes produced by agricultural, mining, industrial, and domestic activities has been caused by the rapid increase of the population, urbanization, and the rise of living standards due to the technological innovations [1]. Thus, use of conventional materials such as bricks, mortar, and cement implies a huge thermal and electric energy consumption caused by the production process with a negative environmental impact. In a circular economic system, the use of by-products for creating new raw materials adds value to the waste, transforming discarded elements into useful new goods [2].

**Figure 1:**
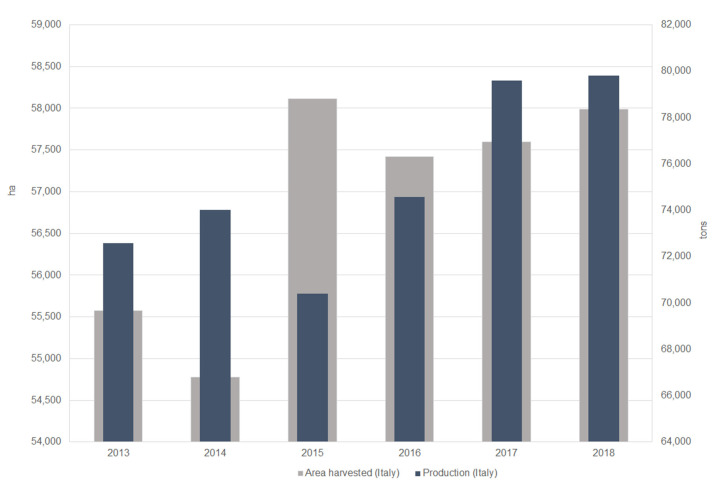
Summary of recent trends in almond production in Italy and harvested area measured in hectares (ha) obtained from elaboration of FAO data [6].

As a consequence, one of the research challenges is to convert waste into new raw matter in order to produce new building components to increase sustainability in the construction sector. Nowadays, a large demand has been placed on agricultural by-products in the development of sustainable building materials. Several research works have studied the potential reuse of aggregates derived from agricultural waste, investigating their efficiency in terms of load-bearing, acoustical, and hygrothermal performances for new ecological materials [3,4,5]. Almond is a crop of major importance worldwide with the production in 2011 being approximately two million tons, according to the FAO [6]. A summary of recent trends in almond production in Italy and harvested area measured in hectares is shown in Figure 1. In Italy the almond industry has different productive configurations, changing according to the management and the mechanization process [7].

A significant increase of the area harvested and the almond production from 2013 to 2018 can be appreciated in Figure 1.

Liu et al. [8] investigated the multisector potential use of agricultural waste in the building construction field, finding different types of final applications: blocks, vegetable biomass for heating, multi-layer solutions, particles, coils, panels for the building envelope. The research on sound absorbing materials has become increasingly important throughout the last decades due to several possible fields of applications, from the aeronautics and road transportation industries to construction and buildings [9]. Currently, the main acoustic absorbing materials on the market have huge costs and represent a big issue in terms of final disposal due to their non-biodegradability [10]. Several researchers studied the potential use of natural components as sound absorbing materials [3,11,12,13]. Fouladi et al. [11] investigated the sound absorption coefficients of different panels made with coir, corn, sugar cane, and dry grass, finding that they are similar to the main common products now existing on the market. Asdrubali et al. [13] built an updated survey investigating the acoustical performances of sustainable materials developed with bio-based and recycled raw materials. They found a great availability on the market and a competitive price.

Martellotta et al. [12] studied the sound absorption properties of olive tree pruning waste with chitosan as a natural binder. They found very interesting performances, measuring absorption coefficients as high as 0.90 for the higher flow resistance sample at frequencies above 1 kHz. Mati-Baouche et al. [3] studied the thermal, acoustical, and mechanical properties of bio-based materials achieved by mixing crushed particles of sunflower stalks with chitosan, a natural binder.

The acoustical properties of a higher porosity composite were tested, achieving a good absorption coefficient.

Furthermore, in order to achieve high energy efficiency buildings, it is fundamental to control the hygric and thermal flux exchange between the internal and external environments. Thus, the use of high performance materials represents an efficient passive technique in lieu of active systems. Keynakli et al. [14] stated that thermal insulation is an essential parameter for building energy saving due to the high potential reduction of the rate of heat transfer. A material is considered as a thermal insulator when its thermal conductivity λ (W·m^−1^·K^−1^) is lower than 0.1 W·m^−1^·K^−1^ [15]. Korjenic et al. [16] developed a new insulating material from jute, flax, and hemp, demonstrating that bio-based materials are comparable to conventional ones for mechanical properties.

Luamkanchanaphan et al. [17] used a blend of narrow-leaved cattail fibers mixed with Methylene Diphenyl Diisocyanate (MDI), developing insulation boards. Thermal conductivity measurements ranged from 0.0438–0.0606 W·m^−1^·K^−1^ for panels with a density of 200–400 kg·m^−3^.

Therefore, in this framework, the development of new technologies based on renewable and natural sources was supported. The current growing interest in almond production derives from the fundamental nutrients constituting the basic fruit [18]. Almond is a fruit with high nutritional value; as a consequence, the industry has restricted the commercial relevance of almonds to the kernel. However, several authors have asserted that the other parts of the fruits (hull, shell, and skin) remain improperly underexplored [18,19].

**Figure 2:**
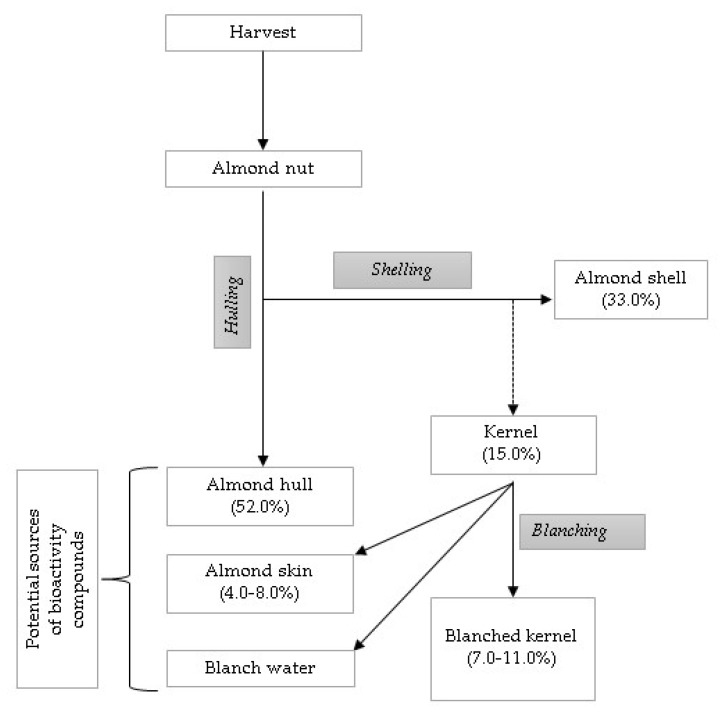
Process of obtaining almond by-products in the almond industry, adapted from Prgomet et al. [19].

Prgomet et al. [19] asserted that during the industrial process of removing the skin, two further by-products in addition to the shell and the hull are produced: blanched skin and blanch water (Figure 2). Thus, 70–85% of the whole almond fruit constitutes residues that currently are scarcely characterized and investigated. The main potential reuse of hulls and shells takes into account the physical and chemical properties of these by-products useful to the pharmaceutical and food sectors. The almond skin consists of carbohydrate polymer (cellulose) and an aromatic polymer (lignin).

Despite the existence of a great number of published papers reporting the main use of almond skin as powder for the production of biofuels like bio-ethanol [20], there are a few studies about the employment of this by-product as reinforcement filler in different thermoplastics [21,22,23]. Mankotia [23] found that in the PA6 plastic (Polyamide 6) the addition of the almond skin powder has positive effects on rheological, thermal, and wear properties. As a result, the valorization of such a by-product as new raw matter for building component production can represent an important way to add value to a low value product.

In order to use natural recycled fibers in the fabrication of porous building materials, a proper choice of the binder is essential. Adhesives play a fundamental role in a great range of areas from packaging to furniture to aerospace technology. The function of an adhesive is to bond separate components by a variety of mechanisms [24]. Most of the adhesives usually include a polymeric substance chemically bonded to the substrate. Heinrich [25] stated that the process and the boundary conditions of application are fundamental as the chemical composition because these factors influence the possible end uses. International standards require the reduction of formaldehyde [26], mainly used in wood furniture, in order to avoid harmful emissions during both the production process and the service life. A large number of researchers identified polyvinyl acetate glue as a suitable bonding material with good adhesive strength [27,28,29]. Ors et al. [28] and Khan et al. [29] focused on the improvement in adhesive strength of porous materials like wood and paper when using polyvinyl acetate glue.

In terms of sustainability, the use of adhesives based on bio and renewable materials can help to promote a circular economy by reducing the carbon footprint [25], even though their overall performance may show some limitations. Gum Arabic is a dried gummy existing on the current market, used mainly as a stabilizer and thickener in foods. However, several researchers described the good properties of gum Arabic, considering multiple industrial uses [30,31]. Because of its physical and mechanical properties, it represents a valid substitute for conventional binders mainly composed of formaldehyde. Rubino et al. [32] successfully used gum Arabic as a binder of wool waste fibers matrix for the production of sound absorbing materials.

In this scenario, the main aim of the present research was to develop new sustainable building composites, suitable as indoor covering materials with high acoustic and hygrothermal performances, using almond skin (AS) waste and two different types of binders: the polyvinyl acetate glue and gum Arabic. A comparison of their performances along with the properties of other building materials with similar bulk density highlighted that the AS materials are promising because of their equivalent or even better performance, compared with the main existing products currently available on the market.

## 2. Materials and Methods

### 2.1. Raw Materials

**Figure 3:**
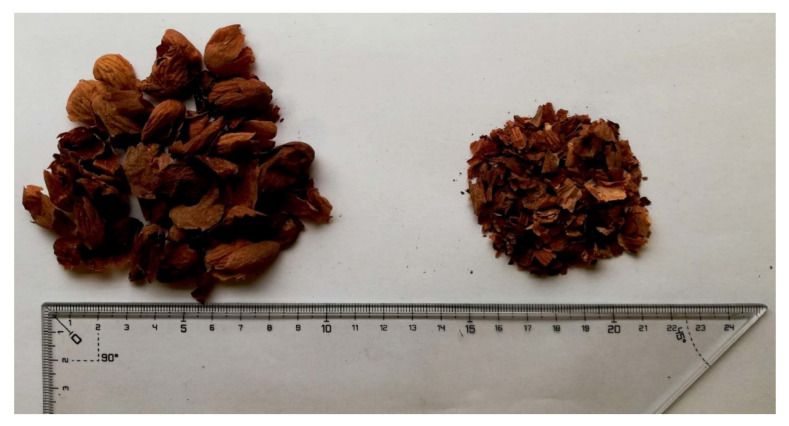
Aggregates used. (On the left) almond residue in its true state used for AS0_GA, (on the right) almond residue in ground state used for AS1_PVA.

Almond skin wastes were used as bio-based aggregates and were provided by ALFRUS srl (Bari, Italy). Two different binders were selected: polyvinyl acetate (PVA) aqueous solution and a biodegradable gum Arabic (GA) aqueous solution (Figure 3).

PVA is a thermoplastic, formaldehyde-free resin available as a water-based emulsion achieved by the polymerization of vinyl acetate monomer; it has a density of 1009 kg·m^−3^. PVA is a low cost, synthetic vinyl polymer with an invisible bond line, prepared by the polymerization of vinyl acetate monomer having the generic formula (C_4_H_6_O_2_)_n_. It is a type of thermoplastic that softens at 30–45 °C. Water emulsions of PVA are used to bind porous materials such as paper, wood, and clothes and to strengthen porous stone. A poor resistance to creep under load has been demonstrated. The clear film produced by the PVA has a great biodegradation resistance and a good resistance to weather, withstanding water, oil, grease, fire. It is mainly adopted as a nonstructural adhesive for projects at room temperature.

Gum Arabic is a complex polysaccharide in the form of natural gum derived from the hardened sap of the Acacia tree. It has a high molecular weight and it is water soluble.

Gum Arabic, a dried exudate from the Acacia Senegal tree, exists in several tropical and semitropical countries and is considered a biopolymer. The aqueous viscosity is strictly related to the origin of the products, the pH level, the electrolyte content, and the pretreatment. The addition of water to gum Arabic results in the reduction of the surface tension, achieving a solution of yellow–red color.

The aggregates were obtained during the manufacturing process of the hulls after peeling the almonds in high temperature water; thus, the thin layer of brown skin was removed and stored in external tanks. Before preparing the mix, the vegetable aggregates were oven-dried at 50 °C until they achieved constant mass ±0.5%, then cooled to environmental temperature in desiccators containing silica gel. The mixtures under study resulted from the heuristic search for the best combination of cohesion between the almond aggregates and the binders and the higher bulk porosity of the final specimens. The first mix, labeled AS0_GA, was based on almond skins used “as is,” mixed with a GA water solution. The average width of the aggregates was 1 cm, the average length was 2 cm and the thickness was 0.5 mm. The second mix, labeled AS1_PVA, included ground almond skin fibers with an average grain size of 3 mm × 3 mm and 0.2 mm of thickness bonded with a PVA water solution.

### 2.2. Sample Preparation

Many elements during the manufacturing process in the laboratory can influence the efficiency of the final samples: the initial water content of the fibers, the particle size distribution of the aggregates, the aggregates/binder ratio, and the type of the binder. Thus, several compositions were preliminarily tested in order to assess the best cohesion between the aggregates and the binders while keeping porosity as high as possible. All the samples were prepared under controlled environmental conditions to ensure that both materials and binders could interact in the best possible way and curing time could be standardized. A water solution was used to mix PVA glue and dissolve GA powder in a magnetic stirrer at normal temperature and relative humidity. The best quantities and ratios resulting from the preliminary tests are shown in Table 1.

**Figure 4:**
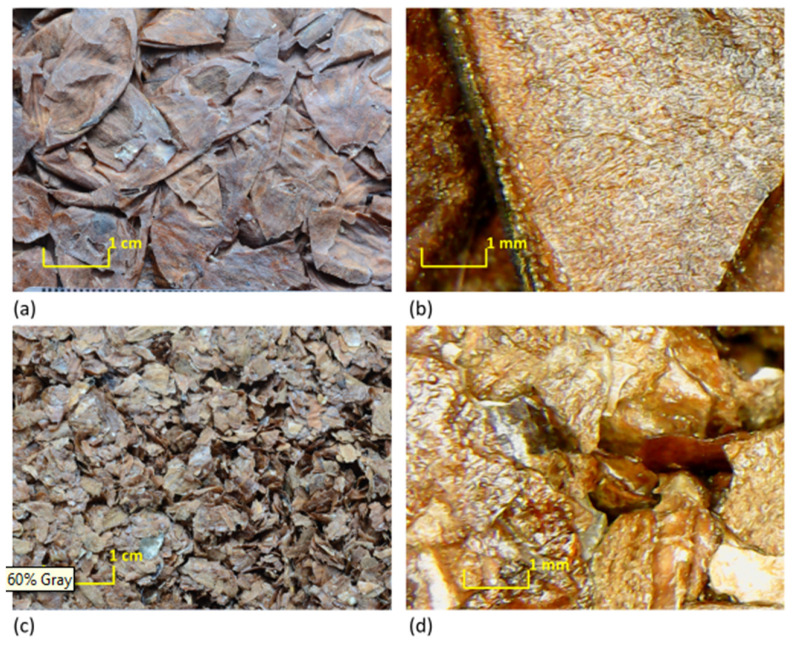
Magnified images of the analyzed samples: (**a**,**b**) AS0_GA; (**c**,**d**) AS1_PVA.

Figure 4 presents the superficial morphology of the specimens. An optical microscope was used to assess the binding effect and to study the interaction between the Gum Arabic and polyvinyl acetate solutions and the aggregates.

The interface between the almond skin and the two different binders is highlighted. A superficial coat due to the binder can be noted on both types of the specimens. The rough surface morphology of the almond skin contributes to enhance the adhesion between the binder and the fiber in composite production, leading to stronger interfacial adhesion.

## 3. Methods

### 3.1. Physical Measurements

According to ISO 12570 [33] the bulk density *ρ_b_* (kg·m^−3^) of the specimens was calculated taking into account the size and the weight of the specimens. Each dimension was the average of three different measurements recorded by an electronic caliper (0.1 mm accuracy); and the weight was registered by an analytical balance (0.01 g accuracy). According to ASTM D4892 [34], the true density *ρ_t_* (kg·m^−3^) of the specimens was measured by an ULTRAPYC 1200-e helium gas pycnometer (Quantachrome, Boynton Beach, FL, USA) in order to determine the bulk porosity *ɛ* as follows:

(1) $$\varepsilon =1-\frac{\rho_{b}}{\delta_{p}}$$

### 3.2. Thermal Measurements

**Figure 5:**
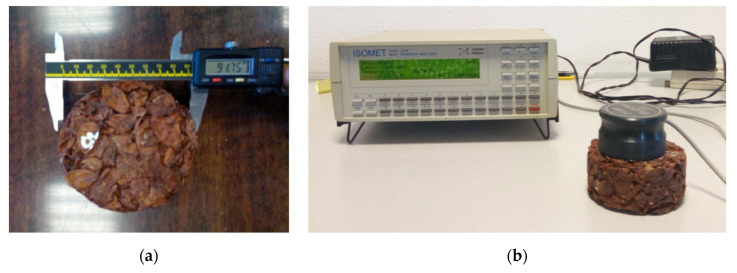
Thermal measurement set up: (**a**) specimen dimensions measurement, (**b**) ISOMET 2104.

The measurement of the dry-state thermal conductivity λ (W·m^−1^·K^−1^), the thermal diffusivity *a* (10^−6^·m^2^·s^−1^), and the volumetric heat capacity *ρc* (J·m^−3^·K^−1^) were performed by an ISOMET 2104 (Applied Precision Ltd., Bratislava, Slovakia), a transient plane source device (Figure 5). The error of the measurement of these thermal parameters was estimated to be within ±4%, ±5%, and ±7%, respectively, according to Bouguerra et al. [35]. For each mix type, three representative samples of 10 cm diameter and 4 cm thickness were taken into account, and the values shown in Table 2 are the mean of three measurements. Before recording the measurements, the specimens were dried in a hot-air oven at 50 °C. Then, all the specimens were stabilized at 23 °C in desiccators containing silica gel.

### 3.3. Hygric Measurements

**Figure 6:**
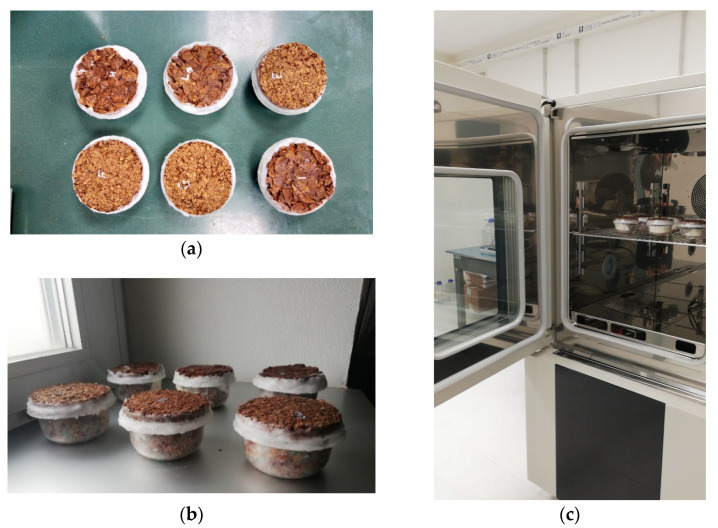
Hygric measurements set up: (**a**,**b**) samples prepared in dry cups; (**c**) Angelantoni DY340 climate chamber.

The hygric parameters of the composites were determined by measuring the water vapor resistance coefficient *µ* (-) according to ISO 12572 [36] in a dry cup. Before starting the test, all the specimens were conditioned at 50 °C in a hot-air oven until achieving a change of mass less than 0.5%. Then, the samples were wax-sealed on the top of transparent vessels containing silica gel as desiccant (Figure 6a,b); an air space of 1.5 ± 0.5 cm was left between the desiccant and the sample. The assemblies were then placed in a temperature- and humidity-controlled environment chamber, Angelantoni DY340 (Angelantoni Test Technologies Srl, Massa Martana PG, Italy), set to 23 °C and 50% RH (Figure 6c).

The mass variations were calculated by recording daily mass using a Mettler PJ300 balance (±0.01 g accuracy, Mettler-Toledo GmbH, Greifensee, Switzerland) until achieving a constant mass loss for unit time. The water vapor resistance coefficient *µ* was then calculated from the experimental data as follows:

(2) $$\mu =\frac{\delta_{a}}{\delta_{p}}$$

where *δ_a_* = 1.94 × 10^−10^ kg·m^−1^·s^−1^·Pa^−1^ is the water vapor permeability of air at 23 °C.

The water vapor permeability, *δ_p_*, of the specimen was estimated from the following Equation:

(3) $$\delta_{p}=\Lambda \cdot d$$

where *d* is the specimen thickness, Λ is the vapor permeance, calculated from Equation (4):

(4) $$\Lambda =\frac{1}{\frac{A\cdot \Delta p}{\Delta G/\Delta \tau }-R_{A}}$$

The vapor pressure gradient Δ*p* is the difference of partial vapor pressure between the air gap in the cup and the air in the climate chamber, *A* is the specimen area, *R_A_* is the water vapor diffusion resistance of the air layer in the cup, and Δ*G*/Δ*τ* is the rate of change in mass.

### 3.4. Acoustic Measurements

**Figure 7:**
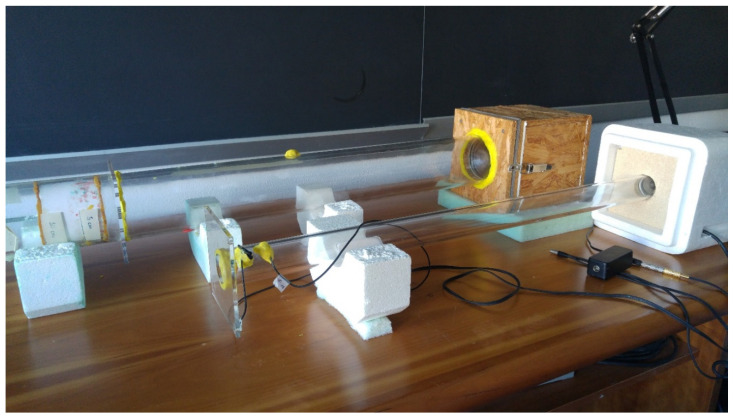
Photograph of the standing wave tubes used for sound absorption at low and medium frequencies (top), and sound absorption at high frequencies and flow resistance measurements (bottom).

Sound absorption measurements were performed by the transfer function method in order to determine the normal incidence sound absorption coefficient according to ISO 10534-2:1998 [37]. Two tubes with different diameters (10 cm, 4 cm) and a thickness of 5 mm were used for the test with the aim to consider the largest spectrum range (Figure 7). Thus, two groups of three specimens were tested, the first one with a 10 cm diameter and the second one with a 3 cm diameter. The tube with an internal diameter of 10 cm had a maximum measurable frequency of 2 kHz, and it used two different microphone distances (6 cm and 20 cm, respectively, yielding a low-frequency limit of 400 Hz and 50 Hz). The emitting end consisted of an 11 cm loudspeaker sealed into a wooden case and suitably isolated from the tube structure by an elastic and protective layer. For the second tube with a diameter of 4 cm the microphone distance was set to 3 cm, and the frequency covered a range between 200 Hz and 5 kHz. In both cases, the sample holder allowed different mounting conditions, close to the rigid termination and at distances that, in the present case, varied between 5 and 10 cm from the wall. All the results were processed by a MATLAB^®^ (2018, Mathworks, Natick, MA, USA) graphical user interface generating a 5 s linear sweep from 70 Hz to 3 kHz, when used in combination with the largest tube, and from 500 Hz to 5 kHz when considering the smallest tube. The performance of the previously described device was compared using the same material (a 5 cm polyester fiber sample) as that of the BSWA SW 260 two-microphone impedance tube, showing that one-third octave band values differed by less than 5% over the entire overlapping spectrum.

### 3.5. Non-Acoustic Measurements

Non-acoustic parameters usually include the descriptors of the porous structure of the material. Their number and type are strongly dependent on the model used to describe the sound absorbing layer. Several motionless skeleton (or “equivalent fluid”) models were available in the literature, spanning from the simplest ones, based on macroscopic empirical models and developed by Delany and Bazley [38] and later improved by Miki [39] (only depending on air-flow resistivity), to the most refined semi-phenomenological and microstructural models. Among them, it was possible to account for the early models reviewed by Attenborough [40], the microstructural model the same author proposed [41], basing the prediction of the acoustical characteristics of rigid fibrous absorbents on five parameters (porosity, flow resistivity, tortuosity, steady flow shape factor, and dynamic shape factor), and possibly others. However, it was the semi-phenomenological model developed by Johnson et al. [42] and subsequently refined by Allard and Champoux [43] that, by using only five non-acoustic parameters (including air-flow resistance *σ*, porosity *ε*, tortuosity *k*_s_, and viscous Λ and thermal Λ’ characteristic lengths), offered a good balance between ease of use and prediction accuracy. Further improvements to the above formulation were given by Lafarge et al. [44], including one more parameter named static thermal permeability k_0′_, and Pride at al. [45], including the static viscous tortuosity α_0_ and the static thermal tortuosity α_0′_.

Considering that only a small subset of the previously mentioned parameters can be directly measured, and that the others need to be estimated according to the procedures described below, the Johnson–Champoux–Allard (JCA) model [42,43] was preferred as the reference one to help in interpreting the results. This model is one of the most frequently used in the literature and is implemented in several vibro-acoustic tools. In addition, the additional parameters required by the more complex models rely on a limited literature background, without considering that the variations they introduce are typically small and refer only to low frequencies. However, just for comparison purposes, results obtained with the Johnson–Champoux–Allard–Lafarge (JCAL) model [44] were also shown for the base cases. Equations used to derive the effective density and the effective bulk modulus according to the proposed models, and mostly derived from Allard and Atalla [46], are described in detail in Appendix A.

Thus, in light of the previously mentioned choice, the non-acoustic parameters considered in this paper were: air-flow resistance *σ*, porosity *ε*, tortuosity *k*_s_, viscous Λ and thermal Λ’ characteristic lengths, and static thermal permeability k_0′_. Among them, porosity was measured directly as described in Section 3.1., as well as air flow resistivity, which was measured according to the acoustic method proposed by Ingard and Dear [47]. According to this setup the sample was located in between two 85 cm long methacrylate tubes (with a 4 cm inner diameter), having a rigid termination on one side and a loudspeaker (Visaton FRS 5, Visaton, Haan, Germany) on the other. The loudspeaker had a flat response between 150 Hz and 20 kHz and was fed by an exponential sine sweep. The pressure drop through the sample was determined by means of two calibrated microphones (Core Sound, Teaneck, NJ, USA) located close to the sample and to the rigid termination allowed to calculate.

As it was impossible to directly measure the remaining parameters, they were estimated. An inverse method [48] was used to estimate the missing ones. Taking advantage of measured absorption coefficients, porosity, and air-flow resistivity, the values of the missing physical properties (i.e., tortuosity, viscous and thermal lengths ratio, and shape factor) were determined by means of a brute-force algorithm developed in MATLAB^®^ to find the set of parameters that allowed the best match between measurements and predictions. Measured parameters were also allowed to vary within their uncertainty ranges, while the properties that were not measurable varied over the entire range of possible values. In particular, tortuosity values between 1 and 6 were explored, while for the shape factor (on which the viscous characteristic length is directly dependent), values between 0.3 and 3.3 were explored. Finally, the ratio of the characteristic lengths varied between 1 and 10, to satisfy the condition that Λ ≤ Λ’. The cost function minimized by the search algorithm was the mean absolute error between measured and predicted one-third octave absorption coefficients in the range from 100 Hz to 3150 Hz.

## 4. Results and Discussion

### 4.1. Thermal and Hygric Characterization

**Figure 8:**
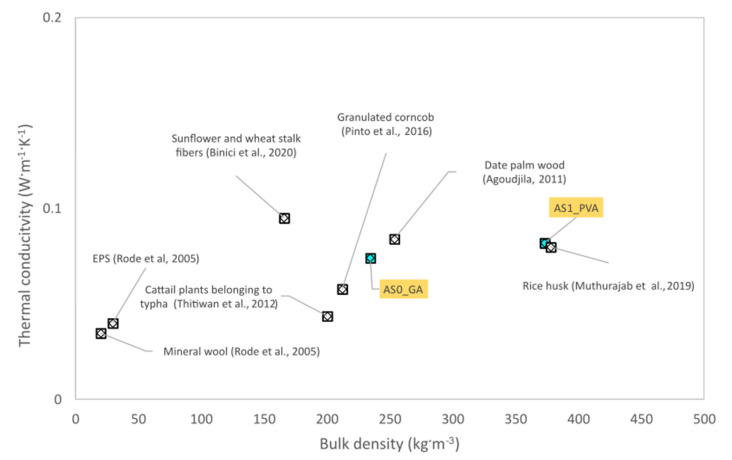
Comparison of thermal conductivity between AS0_GA, AS1_PVA tested samples and different insulators, taken from the literature.

Table 2 shows the results of the thermal properties and hygric measurements executed according to ISO 12570 in a dry cup for the two different blends. In general, a strict correlation between the hygrothermal parameters and the bulk density exists. The reduction of the bulk density corresponds to an increase of porosity and, as a consequence, an enhancement of the thermal parameters and hygric properties. For each mix three representative specimens were evaluated and the final value of the parameters, reported in Table 2, was calculated as the mean of the three measurements. The standard uncertainties were calculated in accordance with JCGM 100:2008 [49]. Both the experimental materials can be considered good thermal insulators as the thermal conductivity was less than 0.1 W·m^−1^·K^−1^ in compliance with Al-Homoud [15]. As shown in Figure 8, the thermal conductivity obtained for the AS0_GA and AS1_PVA samples was lower than 0.2 W·m^−1^·K^−1^; the recorded values were in both cases of the same order as many conventional insulators such as EPS and mineral wool [50]. Furthermore, a good similarity of thermal conductivity could be appreciated in different literature cases referring to the thermal properties of agro-waste materials. Considering the bulk density, the AS1_PVA sample had the thermal conductivity comparable to a rice husk panel [51]. The thermal properties of the AS0_GA specimen were included in the range of typha and date palm wood [17,52,53,54].

In theory, depending on the degree of porosity, the vapor permeability diffusion resistance factor is expected to be lower when a reduction in bulk density occurs. As a matter of fact, AS0_GA had a *µ*-value of 13.036 lower than the water vapor diffusion resistance factor of AS1_PVA.

Comparing the experimental specimens with other sustainable materials available in the literature, it was deduced that the value of the AS material was in accordance with bamboo particleboards, with a *μ*-value ranging from 9.2 and 12.8 [55].

### 4.2. Acoustic and Non-Acoustic Parameters

**Figure 9:**
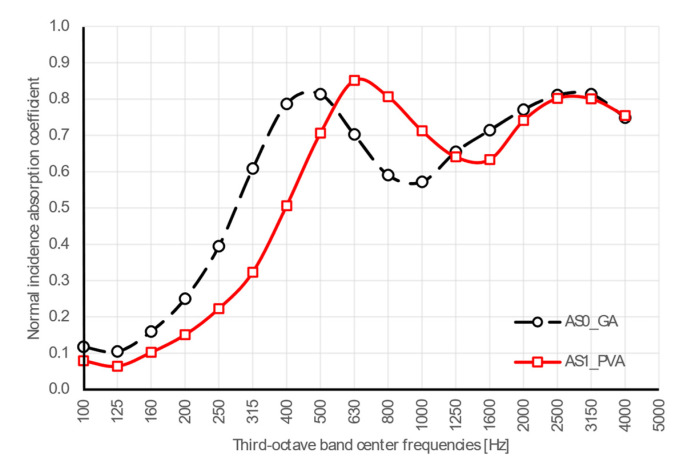
Plot of normal incidence absorption coefficients measured in one-third octave bands for samples with polyvinyl acetate (AS1_PVA) and gum Arabic (AS0_GA) specimens.

The results for normal incidence sound absorption coefficients are shown in Figure 9. The two samples showed nearly the same behavior with two evident peaks spaced out by a drop, which is typical of thick porous materials, although the frequency where the first peak appeared was shifted toward higher frequencies for the AS1_PVA sample. The low frequencies behavior, up to 160 Hz, was rather similar for both cases. The AS0_GA sample sharply increased up to 500 Hz, where the first peak appeared, with α rising up to almost 0.8. Then, a drop appeared with a minimum α value of 0.6, followed by a new peak at 3150 Hz. The AS1_PVA sample showed absorption coefficients lower than the AS0_GA ones up to 630 Hz, where the first peak appeared, with α rising to 0.9; while the AS0_GA curve dropped with α of 0.7. Then, a drop appeared with a minimum absorption value around 0.6, followed by a new peak at 3150 Hz, almost overlapping the second peak of the AS0_GA sample. For porous materials, normal incidence sound absorption is characterized by peaks and valleys, with peaks appearing when particle velocity inside the medium is at a maximum. Assuming that the backing surface is rigid, this first peak takes place at a frequency whose quarter wavelength corresponds to the thickness of the sample and all the others at odd multiples of that frequency [46]. In the present case, for a 5 cm thick panel, the first peak should be at 1.7 kHz, assuming the speed of sound inside the porous medium is the same as that of the air. However, as the first peak was shifted toward lower frequencies in both cases, it was to be expected that, as shown by many authors [3,12,56], due to the more complex and tortuous pore structure, the actual speed of sound was significantly lower. Finally, the subsequent peaks expected at odd multiples of the first peak frequency appeared somewhat smoothed into a single high-frequency peak.

**Figure 10:**
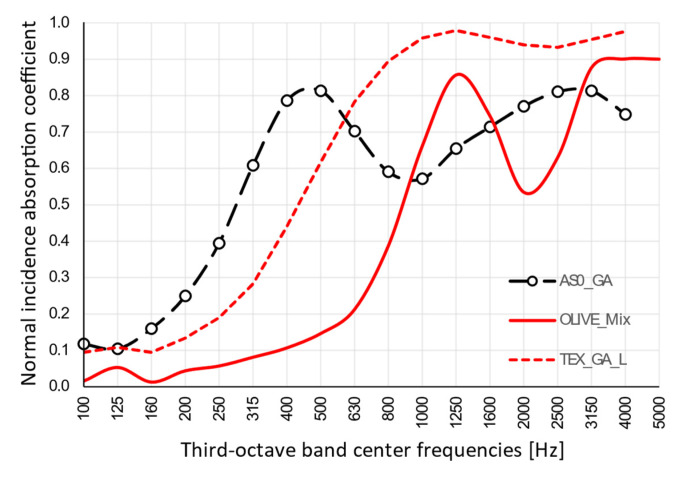
Comparison of measured results for sample with gum Arabic binder (AS0_GA) and data from literature referring to samples made of olive trees (OLIVE_MIX) [12] having same density or specimens with same flow resistivity (TEX_GA_L) [32].

Taking into account Figure 10, a comparison with same-thickness samples made of olive trees [12] with bulk density (between 220 and 240 kg·m^−**3**^) comparable with that of the AS0_GA specimen but with much lower flow resistivity (4.2 kN·s·m^−**4**^), and with recycled textile materials with similar resistivity (14.4 kN·s·m^−**4**^) but different density (93 kg·m^−**3**^), pointed out quite different behaviors, with the almond skin sample showing the first peak significantly shifted toward lower frequencies. Similarity in density, as expected, had very little influence on the results, but even similarity in flow resistivity proved not to be enough to ensure a similar behavior; this suggested that the different first peak positions could be attributed to the major role played by porosity and the morphology of the internal cavities of the specimens.

**Figure 11:**
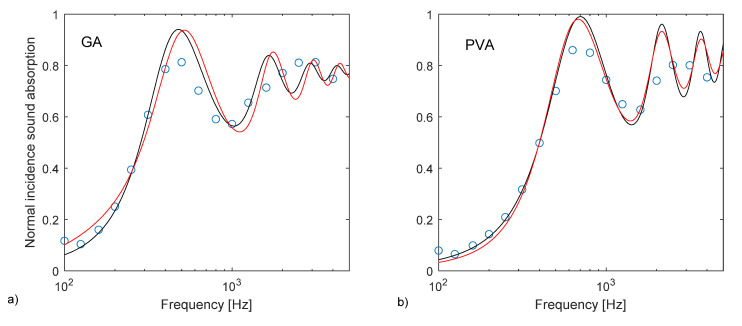
Comparison of measured and best-fit values normal incidence sound absorption coefficients resulting from the application of the JCA model (black curve) and the JCAL model (red curve): (**a**) AS0_GA sample; (**b**) AS1_PVA sample.

**Figure 12:**
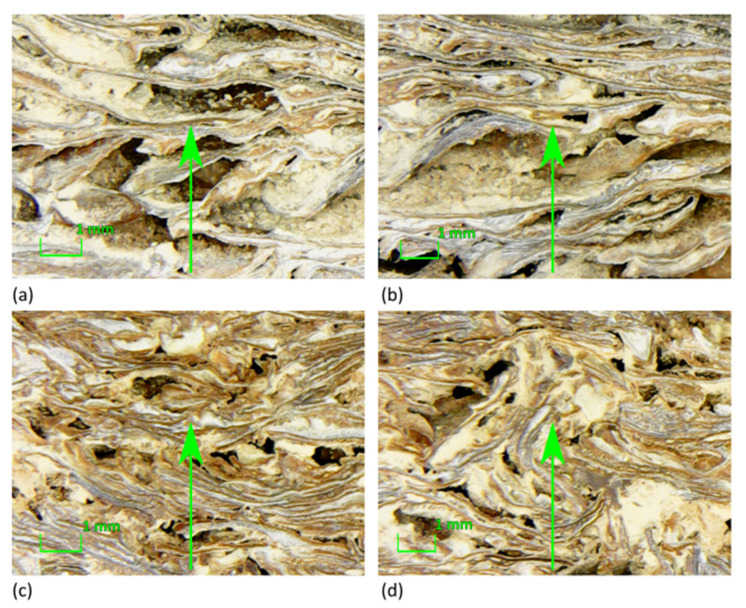
Magnified images of the cross section of the analyzed samples: (**a**,**b**) AS0_GA; (**c**,**d**) AS1_PVA. In all the cases sound propagation takes place along a vertical direction (green arrow).

The application of the phenomenological model defined by Johnson–Champoux–Allard [42,43] and the subsequent indirect determination of the characterizing parameters returned a very good agreement between measured and predicted absorption coefficients, with peaks appearing at the same frequencies, although with slightly different absolute values (Figure 11). The JCAL model [44] was also applied in this case for comparison purposes and apparently provided no significant improvement, compared to the JCA model. In fact, the minimum value of the cost function was slightly higher for the JCAL model than for the simpler one. Predicted speed of sound inside the porous structure was frequency dependent, and its value at the first absorption peak was about 120 m/s for sample AS0_GA and 150 m/s for sample AS1_PVA. The analysis of the parameters resulting from the indirect measure (Table 3) suggested that the multiple layers of thin almond skins originated a very complex channel pattern that resulted in high tortuosity, spanning between 4 and 5.5 values. Such results appear significantly higher than the values typically found in the literature with reference to crushed materials [3,57], which are typically around 3. However, it is possible to find even higher tortuosity values when dealing with open porous asphalt [58]. Following this latter statement, even if high values of tortuosity, according to most empirical formulas [59], are typically associated with very low porosity of the medium, it should be considered that the relationship between porosity and tortuosity is strongly dependent on the shape of the grains that compose the solid matrix, as demonstrated by Turo and Umnova [60]. As reported by Sarradj et al. [58] a general relation between the two variables takes the form of *k*_s_ = ε^−L/(1−L)^, where L is a factor depending on the shape of the particles, varying between 0 when particles are needles parallel to flow, and 1 when particles are disks perpendicular to flow. In the latter case, tortuosity may become very high even when porosity is high. Considering that almond skins, particularly those used “as is,” tend to distribute according to layers mostly perpendicular to the faces exposed to sound (Figure 12), the observed values of tortuosity appear perfectly consistent with that structure. In particular, for AS0_GA the whole skins tended to create many more connected pores and consequently had a lower density and higher porosity. Conversely, AS1_PVA samples had the same layered structure (with some “vertical” inclusions here and there (Figure 12d)), but were much more compact and dense.

Once the origin of the low-frequency shift was clarified, as this may be a desirable feature to obtain in sound absorbing materials that extend their effectiveness range to low frequencies (which are always more difficult to treat while keeping overall thickness within reasonable limits), a further experiment was carried out in order to check consistency of the hypothesis and investigate potential use of the panels under different mounting conditions, which might extend the frequency range in which the panel are efficient.

**Figure 13:**
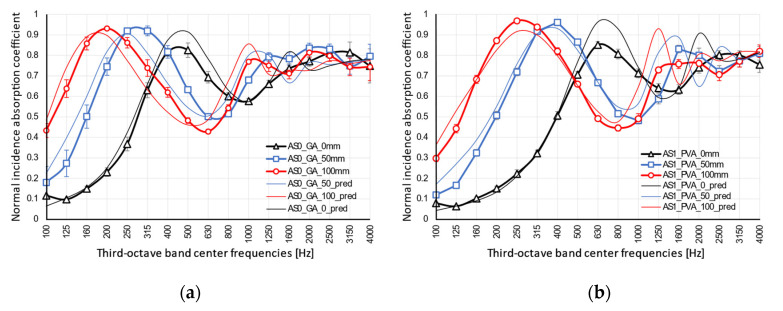
Comparison of measured and predicted normal incidence absorption coefficients resulting from different mounting distances (0, 50, and 100 mm) from hard surface for: (**a**) AS0_GA samples, and (**b**) AS1_PVA samples. Predicted values are based on the application of the JCA model with parameters optimized only for the sample mounted on a rigid surface.

In fact, if the previous interpretation of the shift of the first peak toward low frequency was correct, pointing out the role played by tortuosity and morphology of the porous matrix on the absorption coefficient, mounting the sample at a different distance from the rigid termination should determine a further movement of the peak toward even lower frequencies. Results shown in Figure 13 confirmed that the first peak was shifted exactly according to the to the JCA model predictions (particularly for AS1_PVA samples), although in the high-frequency range the fluctuations between peaks and valleys were less evident than they were in the prediction model.

**Figure 14:**
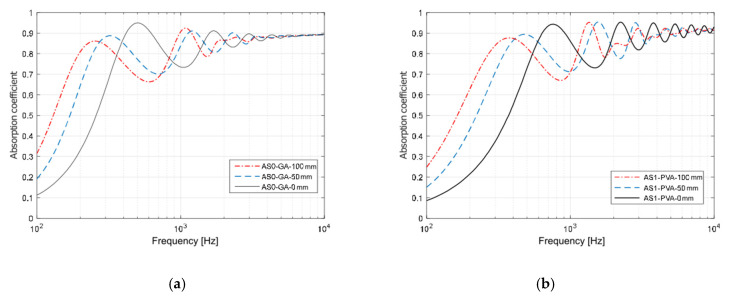
Comparison of diffuse field sound absorption coefficients as a function of frequency predicted, using the JCA model with reference to the sample mounted on a rigid surface and at increasing distances from it. (**a**) Values for sample AS0 with GA binder; (**b**) Values for sample AS1 with PVA binder.

These results suggested that panels made of almond skins, even in the case where they are made by simply mixing them with some sort of binder, may provide a noticeable absorption even in the low-frequency range despite the use of a 5 cm thick panel. The addition of an extra 10 cm air gap (which could be easily obtained in false ceiling application) further contributed to extend the absorption down to 140 Hz for AS0, where a sound absorption higher than 0.7 was found, but in the range between 330 Hz and about 1 kHz values fell again below 0.7. Similar behavior was found for AS1 for which the frequency where the absorption coefficient first reached 0.7 was 160 Hz, while the dip extended from 500 Hz to 1250 Hz. It was interesting to observe that under diffuse field conditions (Figure 14), predicted by calculating a spatially weighted average of the absorption coefficients resulting from different incident angles [46], the dip between the first and second peak appeared significantly attenuated, and the frequency dependent response was generally much smoother. In this case, even using the 5 cm panel without air gap a sound absorption coefficient higher than 0.7 could be obtained from 300 Hz on for AS0_GA and from 500 Hz on for AS1_PVA. Using the 10 cm air gap allowed to push down the frequency where sound absorption reached 0.70 at 170 Hz for the AS0_GA panel and at about 200 Hz for the AS1_PVA panel, thus suggesting that the panels under investigation might be conveniently used as broad band sound absorbers.

## 5. Conclusions

This study focused on a possible way to recycle the almond skin, an agro-waste derived from the almond production process, transforming it into new aggregates used for the production of almond skin materials. Different kinds of tests were performed in order to characterize the hygric, thermal, and acoustical properties of the materials. The results of the experimental campaign allowed to draw the following conclusions.

In general, a good adhesion between the binder and the AS residues was observed, taking into account the morphology and the cross section of the specimens (Figure 4 and Figure 12); the aggregates were well coated and bound together. In particular, for AS0_GA, the whole skins tended to create many more connected pores and consequently had a lower density and higher porosity. Conversely, AS1_PVA samples had the same layered structure (with some “vertical” inclusions here and there) but were much more compact and dense. In fact, although no significant variation of the true density could be appreciated (Table 2), a large variation in the bulk density and bulk porosity *ɛ* was observed. As expected, it was found that lowering the fiber size caused an increase in the bulk density due to less volume being occupied by the almond skin residues at lower sizes.

Considering the thermal properties, it was found that, due to the thermal conductivity less than 0.1 W·m^−1^·K^−1^, both AS0_GA and AS1_PVA samples behaved as good insulators. In addition, the thermal conductivity of both the samples was comparable to the thermal performance of other agro-waste materials and of widespread insulators like EPS and mineral wool. The hygric properties of the experimental specimens, as well, were in the range of bamboo particleboards with a *μ*-value between 9.2 and 12.8.

From the acoustic point of view, the results of the normal incidence sound absorption coefficients measurement showed that both the samples outperformed the typical behavior of thick porous materials. In fact, for typical porous materials, peak location was strongly dependent on the thickness of the sample. In this case it was expected at 1.7 kHz, significantly above the actual observed location. This result was explained in terms of a more complex and tortuous morphology of the pore structure of the specimens.

A very good agreement between measured and predicted absorption coefficients, with peaks appearing at the same frequencies, was achieved using the phenomenological model defined by Johnson–Champoux–Allard [38]. Some parameters were indirectly measured and a tortuosity ranging between 4 and 5.5 was found, in good agreement with the complex channel pattern visible in the cross-section images (Figure 12). In particular, for the AS0_GA specimen it was observed that layers mostly perpendicular to the faces (and to sound propagation) were created. This justified the observed values of tortuosity, perfectly in accordance with that structure.

The previously described experimental study broadly supported the use of almond skin waste to manufacture innovative and sustainable building materials. The physical, hygrothermal, and acoustic properties demonstrated the high potential for such building components.

Further investigations are underway to assess the mechanical performances and the fire protection properties, moreover, evaluating the suitability of other sustainable and eco-friendly binders to be used with the almond skin waste. Furthermore, an LCA analysis and a techno-economic feasibility study for the induction of a building panel on market are necessary and they will be carried out to support future potential commercial applications.

## Figures and Tables

**Table 1:** Details of the different mix designs.

Solution	Almond Skin (A) (g)	Gum Arabic (B) (g)	Polyvinyl Acetate (B) (g)	Water (g)	B/A Ratio
AS0_GA	480	150	-	750	0.30
AS1_PVA	480	-	300	160	0.62

**Table 2:** Summary of hygrothermal properties for each mix. Values in brackets represent standard deviations of measured values.

Mix Code	Bulk Density	True Density	Thermal Conductivity	Thermal Diffusivity	Volumetric Heat Capacity	Water Vapor Resistance
	*ρ_b_*	*ρ_t_*	*λ*	*α*	*ρc*	*µ*
	(kg·m^−3^)	(kg·m^−3^)	(W·m^−1^·K^−1^)	(10^−6^ m^2^·s^−1^)	(10^6^ J·m^−3·^K^−1^)	(-)
AS0_GA	234.65 (13.99)	1255.29 (19.88)	0.074 (0.0035)	0.180 (0.0276)	0.416 (0.0533)	13.0 (0.2)
AS1_PVA	373.10 (6.20)	1162.58 (7.29)	0.082 (0.0080)	0.219 (0.0342)	0.384 (0.0745)	14.9 (1.4)

**Table 3:** Summary of non-acoustical parameters measured (underlined) or indirectly derived from the application of the JCA (italicized) and JCAL models. Values in brackets represent standard deviations.

Sample Code	Model	Bulk Porosity ɛ	Air-flow Resistivity σ	Tortuosity	Shape Factor	Ratio of Characteristic Dimensions	Static Thermal Permeability
		(-)	(kN·s·m^−4^)	(-)	(-)	(-)	(m^2^)
AS0_GA	JCA	0.81 (0.003)	13.377 (1.164)	*5.50*	*3.00*	*1.50*	
	JCAL			5.40	3.00	2.00	3.1 × 10^−9^
AS1_PVA	JCA	0.68 (0.002)	23.371 (1.791)	*4.40*	*1.36*	*1.95*	
	JCAL			5.00	2.36	1.80	2.1 × 10^−9^

## Appendix A

In order to calculate the sound absorption coefficient according to the JCA model, the dynamic (or effective) density and bulk modulus are requested. The first is based on the work by Johnson et al. [42], where visco-inertial dissipative effects inside the porous media are described, and is given by:

(A1) $$\rho_{e}=k_{s}\rho_{0}\left[1+\frac{\sigma \varepsilon }{j\omega \rho_{0}k_{s}}\sqrt{1+j\frac{4k_{s}^{2}\eta \rho_{0}\omega }{\sigma^{2}\Lambda^{2}\varepsilon^{2}}}\right]$$

where *ρ*_0_ is the static density, *k*_s_ is the tortuosity, *σ* is the flow resistivity, *ε* is the porosity, *ω* is the angular frequency, *η* is the dynamic viscosity, Λ is the viscous characteristic length and *j* is the complex unit.

The work by Champoux and Allard [43] is used to describe the thermal dissipative effects and calculate the dynamic bulk modulus according to the formula:

(A2) $$K_{e}=\frac{\gamma P_{0}}{\gamma -\left(\gamma -1\right)/\left(1+\frac{8\eta }{j\Lambda {’}^{2}N_{p}\rho_{0}\omega }\sqrt{1+j\frac{\Lambda {’}^{2}N_{p}\rho_{0}\omega }{16\eta }}\right)}$$

where γ is the ratio of the specific heat capacities, *P*_0_ is the atmospheric pressure, *N*_p_ is the Prandtl Number, and Λ’ is the thermal characteristic length.

Finally, according to Lafarge et al. [44], the dynamic bulk modulus is modified to account for low-frequency thermal effects, yielding the following formula:

(A3) $$K_{e}=\frac{\gamma P_{0}}{\gamma -\left(\gamma -1\right)/\left(1+\frac{\varepsilon \eta }{jk_{0}N_{p}\rho_{0}\omega }\sqrt{1+j\frac{4k_{0}^{2}N_{p}\rho_{0}\omega }{\eta \Lambda {’}^{2}\varepsilon^{2}}}\right)}$$

where k_0_ is the static thermal permeability.

Once the dynamic density and bulk modulus have been determined it is possible to calculate the characteristic impedance *z*_c_ as

(A4) $$z_{c}=\sqrt{K_{e}\rho_{e}}$$

and the propagation wave number as

(A5) $$k=\omega \sqrt{\frac{\rho_{e}}{K_{e}}}$$

so that the absorption coefficient may be calculated according to the usual formulas, as well as other important properties like the actual dynamic speed (frequency dependent) that is given by ω/k.
